# Abnormal muscle in the anterior compartment of the forearm: a case report

**DOI:** 10.1186/1757-1626-2-9125

**Published:** 2009-12-02

**Authors:** Vincent Rodrigues, Satheesha B Nayak, Mohandas KG Rao, Venkataramana Vollala, Nagabhooshana Somayaji, Ashutosh S Rao

**Affiliations:** 1 Department of Anatomy, Faculty of Medicine, AIMST University, Semeling, Bedong- 08100, Kedah, Malaysia; 2 Department of Anatomy, Melaka Manipal Medical College, Manipal, 576 104, India; 3 Department of Orthopedics, Faculty of Medicine, AIMST University, Semeling, Bedong- 08100, Kedah, Malaysia

## Abstract

During routine dissection for the undergraduate medical students, we encountered an unusual, additional muscle in the anterior compartment of the forearm. This muscle took origin from the anterior surface of the radius in common with the flexor digitorum superficialis muscle. It had a tendon of origin and a tendon of insertion. Its fleshy radial belly and the tendon of insertion, crossed superficial to the median nerve. The muscle was inserted partly to the flexor retinaculum and partly to the undersurface of palmar aponeurosis. The observations made by us in the present case will supplement our knowledge of variations of the muscles in this region which could be useful for hand surgeons as it could possibly compress the median nerve because of its close relationship to it.

## Introduction

Variations in muscles of the extensor compartment of the forearm are quite common. However, in the flexor compartment not many variations are noted and occurrence of an additional muscle is very uncommon. Normally, the anterior compartment of the forearm has 5 superficial and 3 deep muscles.

The superficial muscles are pronator teres, flexor carpi radialis, palmaris longus, flexor digitorum superficialis (FDS) and flexor carpi ulnaris and they take a common origin from the medial epicondyle of the humerus. However, some of these muscles like pronator teres and flexor carpi ulnaris have additional origins from the ulna and flexor digitorum superficialis has additional origins from parts of both ulna and radius. The deep muscles are flexor digitorum profundus (FDF), flexor pollicis longus and pronator quadratus and they have separate origins from the different parts of radius and ulna [1].

The median nerve is formed in the axilla by the union of its medial and lateral roots. It passes through the anterior compartment of the arm, cubital fossa and then through the anterior compartment of the forearm. It enters the palm by passing through the carpal tunnel. In the forearm, it passes between the two heads of the pronator teres muscle and later through the plane between the FDS and FDP muscles. About an inch proximal to the flexor retinaculum it comes out of this plane and travels just posterior to the distal part of tendon of palmaris longus [1]. We are reporting a rare case where an additional muscle was found in the anterior compartment of the forearm in addition to the above mentioned muscles.

## Case presentation

**Figure 1:**
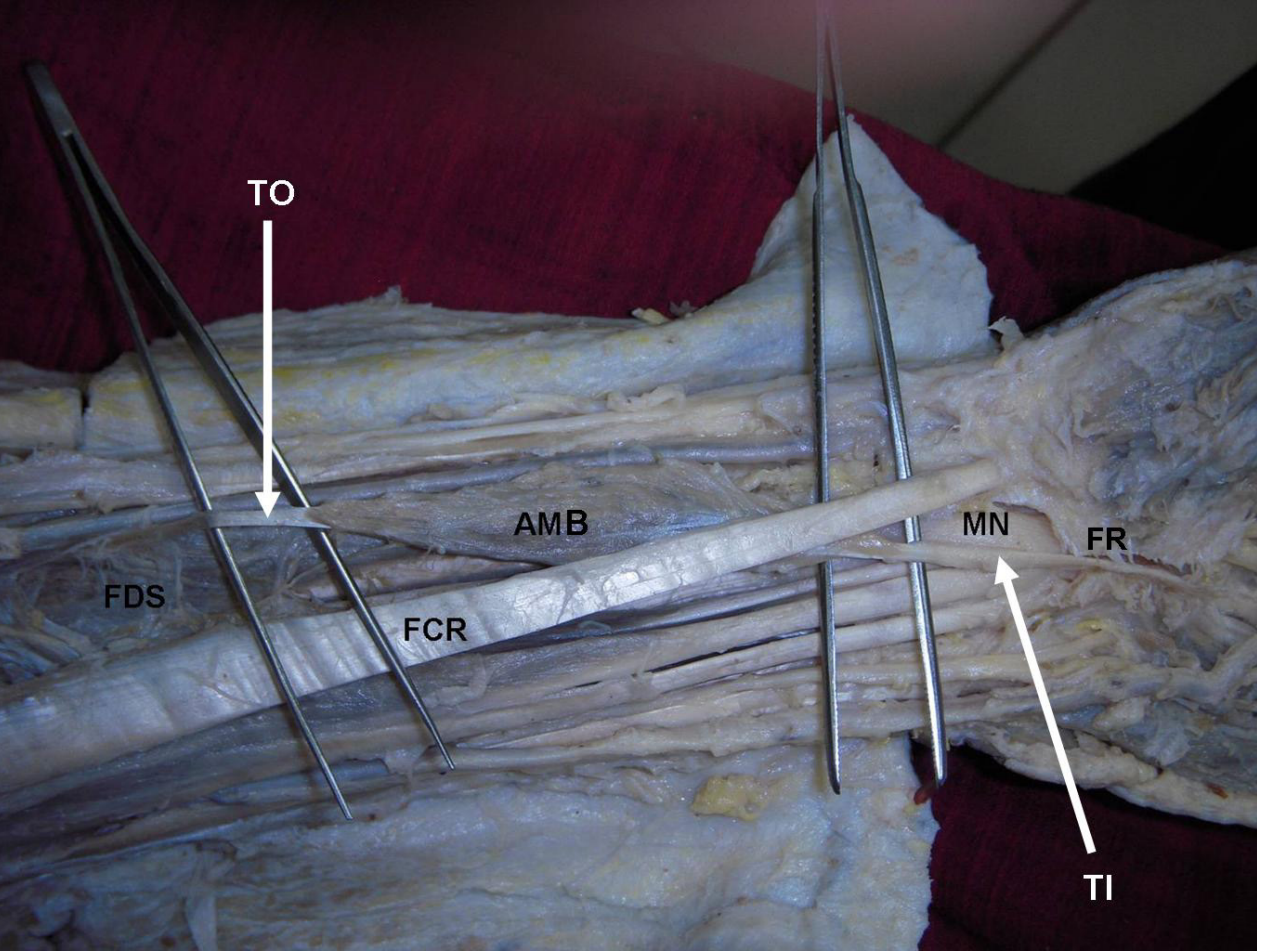
**Photograph of dissection of the distal part of the anterior compartment of the forearm and palm**. The picture shows the exposed anterior compartment of the forearm and proximal part of the palm to show the additional muscle belly (AMB) with two tendons, a tendon of origin (TO) and a tendon of insertion (TI). The flexor retinaculum (FR) has been split to expose the carpal tunnel and the tendon of insertion (TI) has been lifted with a forceps. FDS- flexor digitorum superficialis; FCR- flexor carpi radialis, MN- median nerve.

**Figure 2:**
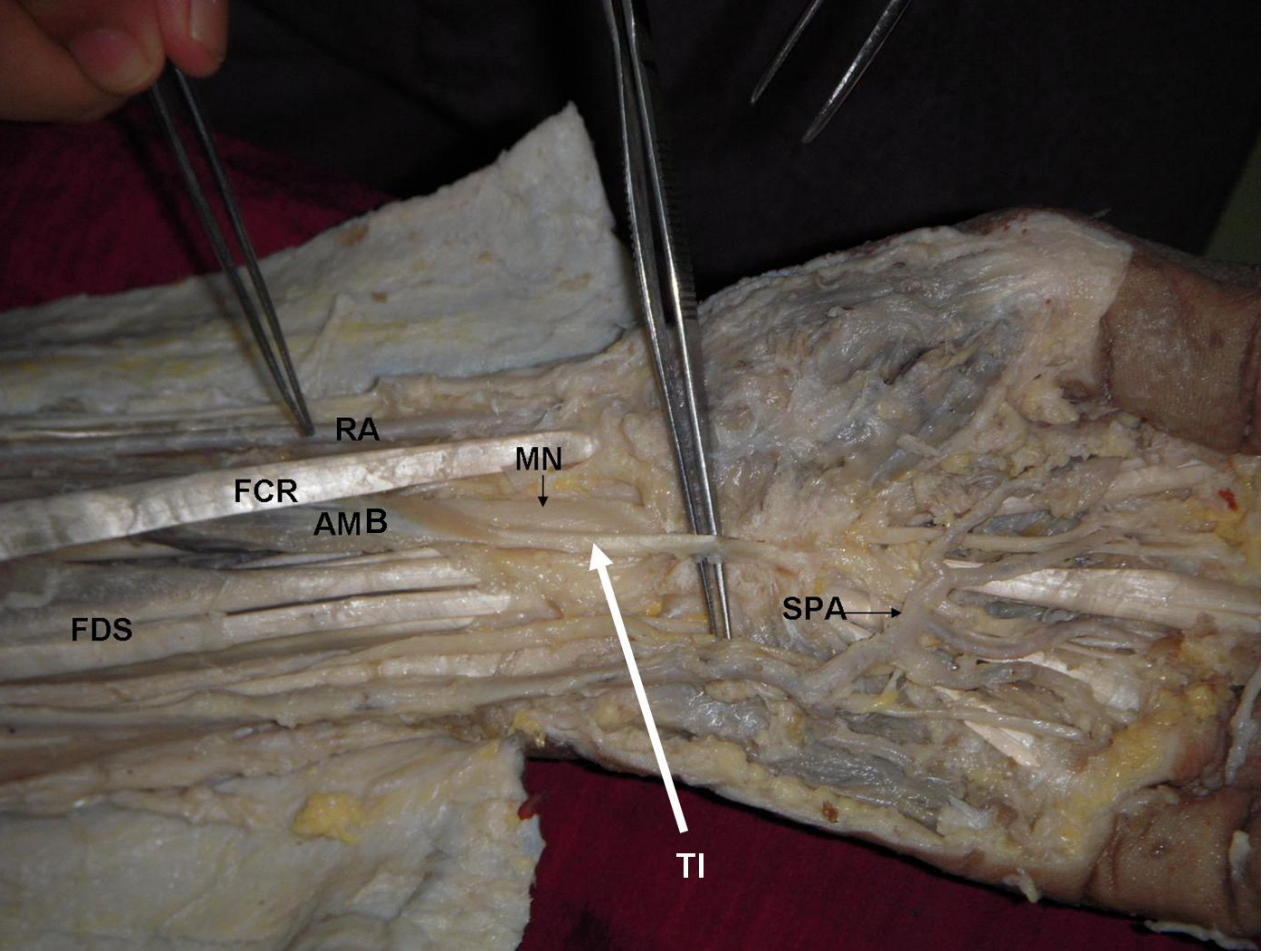
**The picture shows the exposed anterior compartment the forearm and palmar region to show the additional muscle belly (AMB) and its tendon of insertion (TI) getting attached to the palmar aponeurosis and flexor retinaculam**. FDS- flexor digitorum superficialis; FCR- flexor carpi radialis, MN- median nerve, RA- radial artery; SPA- superficial palmar arch.

In a South Indian cadaver aged approximately 60 years, during routine dissection for undergraduate medical students, we found an abnormal muscle in the anterior compartment of the left forearm. The anomaly was unilateral. The muscle had a fleshy belly of about 5 inches and two tendons at its two ends; the tendon of origin and the tendon of insertion. The tendon of origin was attached to the anterior surface of the radius in common with the radial origin of the FDS muscle (Figure 1 and Figure 2). The fleshy part of the muscle and the long tendon of insertion crossed superficial to the median nerve from lateral to the medial side (Figure 1 and Figure 2). The tendon was partly inserted to the deeper surface of flexor retinaculum and then flattened out and merged with the undersurface of the palmar aponeurosis. It was interesting to note that the long tendon of insertion was passing through the carpal tunnel as it entered the palm from the forearm. The muscle was deriving its innervation by a branch of median nerve.

## Discussion

Presence of the abnormal muscles in the flexor compartment of the forearm is not very common. In most cases, these muscles go unnoticed as they do not produce any symptoms in the individual. These abnormal muscles may cause functional deficits by compressing the neurovascular structures. In such cases, a surgical intervention is needed. There are several reported variations of the FDS muscle. Ronald et al [2] have listed a series of variations of flexor digitorum superficialis. Some of the variations presented by them are as follows

1. Absence of the tendon for the little finger

2. Suppression of the radial origin

3. A deep slip to the annular ligament, coexisting with palmaris longus

4. A doubled coronoid origin-the upper slip fleshy, the lower one tendinous

5. A connecting muscular band between the origins of the flexor sublimis and flexor pollicis longus

6. A slip from this muscle to the palmar fascia coexisting with a feeble palmaris longus

A case of split FDS has been reported by Shoja et al [3]. In this case, the median nerve passed between the two bellies. In extremely rare cases, a muscle called radiopalmaris arises from the anterior surface of radius in common with FDS and gets inserted to the palmar aponeurosis or to the synovial sheath covering the FDS [2]. The muscle that we found is almost similar to the radiopalmaris reported earlier. However, in our case the muscle also had insertion to the flexor retinaculum.

An abnormal muscle may simulate a ganglion [4,5] or a soft tissue tumor [6,7] or if in close proximity to a nerve, it may cause pressure neuritis and produce symptoms such as a carpal tunnel syndrome [8]. The muscle that we are reporting might cause any of such problems. On the other hand, this muscle may add to the function of the palmar aponeurosis. It might give additional protection for median nerve during carpal tunnel release.

## Clinical significance of aberrant tendons in the carpal tunnel

The most established clinical significance of presence of aberrant tendons and muscle in the forearm and the carpal tunnel is the resultant compression of the median nerve leading to the clinical entity of carpal tunnel syndrome. Entin [9] grouped causes of carpal tunnel syndrome into three categories: those reducing the capacity of the tunnel; those increasing the volume of its contents and those that form part of a systemic condition [10]. Presence of aberrant or additional muscles located in the carpal tunnel (referred to as space occupying lesions in the carpal tunnel) has been blamed for symptoms of carpal tunnel syndrome. Investigation tools used in the diagnosis of causes for carpal tunnel syndrome prior to surgery includes ultrasonographic studies of the carpal tunnel and MRI studies to rule out soft tissue causes for the symptoms [11]. Wrist is a common site for tendon injuries and presence of aberrant tendon in this region can mislead the surgeons during identification and repair of such injuries [12]. Palmaris longus is considered to be highly variable in its presence. It can be absent in 3 to 24% of the population [13-15]. Absence of the palmaris longus is also said be associated with other anatomical abnormalities in the hand. The relationship of the presence of the palmaris longus to occurrence of carpal tunnel syndrome has been reported [16]. Hence In individuals where the palmaris longus is absent it may be hypothesized that anatomical variations may present as presence of aberrant muscles or tendons leading to the occurrence of carpal tunnel syndrome. Presence of abnormal tendons in the carpal tunnel has also been cited as one of the contraindications to endoscopic carpal tunnel release of the tunnel [17].

## Conclusion

We would like to conclude by stating that the observations made by us in the present case will supplement our knowledge of variations of the muscles in this region which could be useful for hand surgeons as it could possibly compress the median nerve because of its close relationship to it.

## Consent

Written informed consent was obtained from the subject's relative for publication of this case report.

## Competing interests

The authors declare that they have no competing interests.

## Authors' contributions

VR discovered the variation and did the dissection. He also obtained written consent from the other authors and also partially involved in the literature search. SBN and Mohandas MKGR wrote the case report and drafted the manuscript. They were also partially involved in the literature search. VV and NS did a detailed literature search and helped draft the manuscript. AS gave input on the clinical and surgical importance of the case.
